# Case Report: “Incognito” proteus syndrome

**DOI:** 10.12688/f1000research.13993.1

**Published:** 2018-02-26

**Authors:** Michelangelo Vestita, Angela Filoni, Nicola Arpaia, Grazia Ettorre, Domenico Bonamonte

**Affiliations:** 1 Section of Plastic and Reconstructive Surgery, Department of Emergency and Organ Transplantation, University of Bari, Bari, 70124, Italy; 2 Section of Dermatology, Department of Biomedical Sciences and Clinical Oncology, University of Bari, Bari, 70124, Italy; 3 Section of Dermatology, Miulli Regional Hospital, Bari, 70021, Italy

**Keywords:** proteus syndrome, cerebriform, keloid, diagnosis, elusive, hidden

## Abstract

Proteus syndrome (PS) is a postnatal mosaic overgrowth disorder, progressive and disfiguring. It is clinically diagnosed according to the criteria reported by Biesecker *et al*.

We describe the case of a 49-year-old woman who presented with a 10-year history of pauci-symptomatic infiltrating plaque lesions on the sole and lateral margin of the left foot, which had been diagnosed as a keloid. The patient had a positive history for advanced melanoma and a series of subtle clinical signs, such as asymmetric face, scoliosis, multiple lipomas on the trunk, linear verrucous epidermal nevi, and hyperpigmented macules with a mosaic distribution. Even if the clinical presentation was elusive, she had enough criteria to be diagnosed with PS.

This case describes the first evidence, to the best of our knowledge, of pauci-symptomatic PS in adulthood, reports its rare association with advanced melanoma, and illustrates the importance of even minor cutaneous clinical signs, especially when atypical, in formulating the diagnosis of a complex cutaneous condition such as this.

## Introduction

Proteus syndrome (PS) is a postnatal mosaic overgrowth disorder, which was originally described by Cohen and Hayden in 1979 ^1^. In 1983, the syndrome was named after a minor Greek deity who had the power to change his appearance ^2^. The occurrence of this disorder is sporadic, with a prevalence of less than one per million ^3^. PS is a progressive, disfiguring disorder caused by a somatic point mutation in *AKT1* leading to gene activation. The product of this gene is involved in cell proliferation and apoptosis suppression, acting through the mammalian target of rapamycin signaling pathway, which may explain the overgrowths in this syndrome ^4^. Clinically, this disorder is characterized by typically asymmetric, disproportionate, postnatal overgrowth of tissues derived from any of the three germ layers. While skin, bone, connective, and adipose tissues are most commonly involved, some patients present with overgrowths of the central nervous system, spleen, thymus, or colon. In addition, patients may also present with a range of tumors, pulmonary complications, and a striking predisposition to deep vein thrombosis and pulmonary embolism ^5^. PS is clinically diagnosed according to the criteria described by Biesecker *et al*. ^6^ ( Table 1).

**Table 1.** Diagnostic criteria for Proteus syndrome ^6^.

**General** **criteria**	▪ Mosaic lesions ▪ Sporadic disease ▪ Progressive disease
**Category A**	▪ Cerebriform connective tissue nevus
**Category B**	▪ Epidermal nevus (linear verrucous epidermal nevus) ▪ Overgrowth of various body tissues ▪ Tumors (bilateral ovarian cystadenomas or parotid monomorphic adenomas)
**Category C**	▪ Dysregulated adipose tissue (lipomas and regional lipo-hypoplasia) ▪ Vascular malformations (capillary malformations, venous malformations, and/or lymphatic malformations) ▪ Characteristic facial phenotype (long face, minor downward slanting palpebral fissures, low nasal bridge, wide nares, and open mouth at rest).

## Case

**Figure 1.**
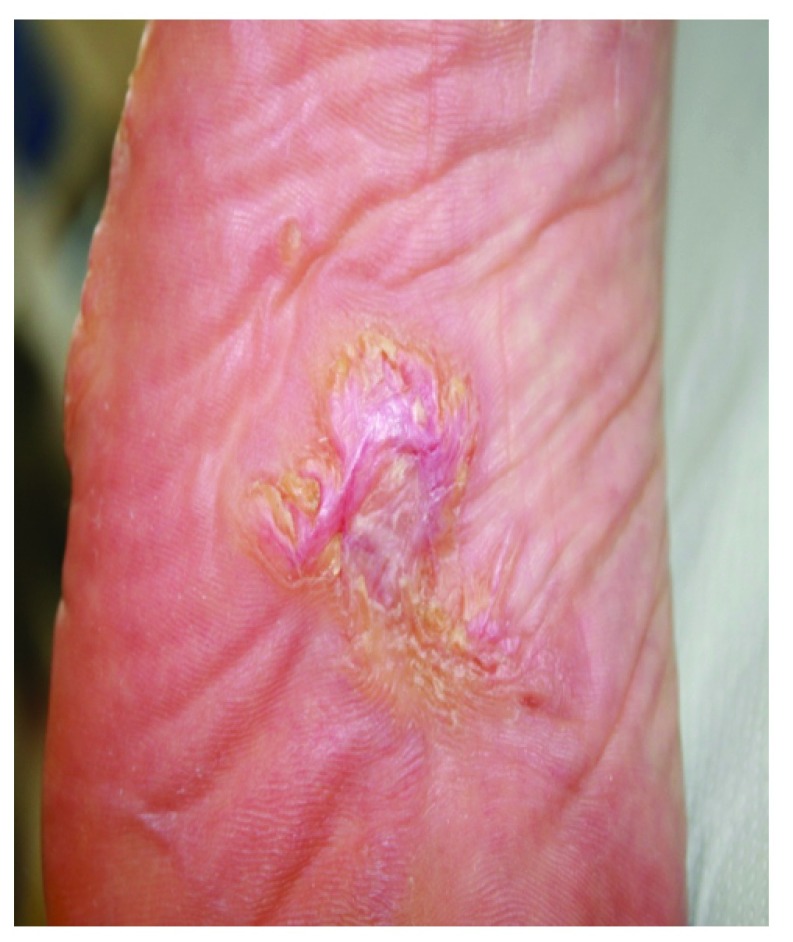
Proteus syndrome: Modestly developed connective tissue nevus of the left foot, previously misdiagnosed as a keloid.

**Figure 2.**
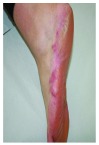
Proteus syndrome: Lateral view of the affected foot.

**Figure 3.**
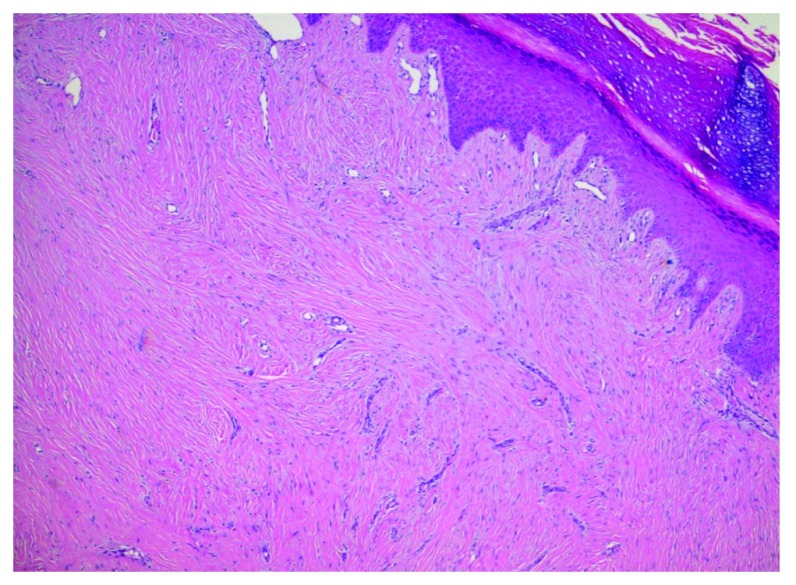
Proteus syndrome: Biopsy from the cerebriform nevus of the foot. Hematoxylin and eosin (magnification x100).

A 49-year-old woman presented with a 10-year history of pauci-symptomatic infiltrating plaque lesions on the sole and lateral margin of the left foot ( Figure 1 and Figure 2). The lesions simulated and had been misdiagnosed as keloids, but there was no history of trauma to the area. The patient reported that similar lesions had affected her great-grandmother. The patient had a positive history for stage IV melanoma, and she had finished chemotherapy ^7^ just 3 months before our observation. Physical examination also revealed multiple lipomas on the trunk, linear verrucous epidermal nevi, and hyperpigmented macules with a mosaic distribution. Additionally, she presented with an asymmetric face, dysmorphic skull with frontal-parietal hyperostosis, dropped shoulders, scoliosis, and a stiff spine. Her legs were asymmetric with disproportionate overgrowth, the left leg being longer than the right one and having ectatic veins. In addition, computed tomography documented uterine fibromas, and abdominal magnetic resonance imaging demonstrated hepatic angiomatosis. A skin biopsy specimen from the left foot stained with hematoxylin and eosin revealed remarkable hyperkeratosis, epidermal hyperplasia, dermoepidermal fibrosis with extensive sclerosis of the reticular dermis, thickened collagen bundles, and fat-cell entrapment ( Figure 3). We made the diagnosis of Proteus syndrome. No therapeutic intervention was carried out.

## Discussion

To meet the diagnostic criteria for PS, a patient must fulfill all three general criteria, plus a single criterion from category A or two criteria from category B or all three criteria from category C ( Table 1). Even though our patient had not previously sought medical care, when she presented to us her condition fulfilled the criteria, and the diagnosis of PS was confirmed.

This case illustrates the importance of even minor cutaneous clinical signs, especially when atypical. They should not be overlooked because, together with other clinical and diagnostic findings, they may lead to the diagnosis of a specific condition. This is especially true in mosaic diseases, such as PS, in which the wide variety of tissue types and cells that are involved may not be apparent at the first examination. Subtle cutaneous forms of PS have been described in infants ^4^, but to the best of our knowledge, this is the first case in which the cutaneous signs remained elusive in adulthood. We do not know whether the chemotherapy she had been administered had somehow altered the lesion morphology ^7^, but that seems unlikely, as that occurred in adulthood and the patient referred no significant changes in shape and volume.

Correct recognition of pauci-symptomatic “incognito” PS is essential, as PS patients are known to be exposed to a higher risk to develop tumors ^1– 4^, such as meningiomas, breast and ovarian cancer, parotid adenoma, and others. We do not know whether melanoma occurrence is facilitated by PS, and the literature provides scarce data on this. The association between PS and melanoma in this case is either a novel finding or an incidental coexistence. At present our patient reached the 24 months follow up with no clinical or instrumental signs of recurrence.

## Consent

Written informed consent for publication of their clinical details and clinical images was obtained from the patient.
