# Supplementary material for: Case Report: “Incognito” proteus syndrome
Source: F1000Res. 2018 Feb 26;7:228. [Version 1] doi: 10.12688/f1000research.13993.1 (PMC5843845; doi:10.12688/f1000research.13993.1)
Supplement: Peer review history

# Peer review: Referee response for version 1

**DOI:** 10.5256/f1000research.15209.r31219

**Published:** 2018-03-08

**Authors:** Giuseppe Micali

**Affiliations:** 1 Dermatology Clinic, University of Catania, Catania, Italy

It is an interesting and well-presented case report of pauci-symptomatic, previously overlooked Proteus syndrome in a 49-year-old woman. Proteus syndrome is a rare, complex disorder with multisystem involvement and remarkable clinical variability. The authors highlight the relevance of the recognition of minor cutaneous clinical signs for the diagnosis of minimal forms of the disease, as several complications, some of which life-threatening, may potentially occur in these patients.

I have read this submission. I believe that I have an appropriate level of expertise to confirm that it is of an acceptable scientific standard.

# Peer review: Referee response for version 1

**DOI:** 10.5256/f1000research.15209.r31217

**Published:** 2018-02-28

**Authors:** Marco Marcasciano

**Affiliations:** 1 Unit of Plastic, Reconstructive and Aesthetic Surgery, Sapienza University of Rome, Rome, Italy

Proteus syndome is a complex multi-organ mosaic disorder, characterized by progressive overgrowth and disfiguring. The authors did a nice job in describing the occurrence of such a multi-faceted condition in its (rare) minimal symptomatic presentation. To suspect and correctly diagnose a Proteus in view of (apparently) so few clues is indeed challenging. Since the repercussions are relevant (increased incidence of malignancies) the educational message conveyed here is an important one: never underestimate the importance and significance of a given set of signs and symptoms, as subtle as they may be.

I have read this submission. I believe that I have an appropriate level of expertise to confirm that it is of an acceptable scientific standard.
